# Caveolae-Dependent and -Independent Uptake of Albumin in Cultured Rodent Pulmonary Endothelial Cells

**DOI:** 10.1371/journal.pone.0081903

**Published:** 2013-11-27

**Authors:** Hui-Hua Li, Jin Li, Karla J. Wasserloos, Callen Wallace, Mara G. Sullivan, Philip M. Bauer, Donna B. Stolz, Janet S. Lee, Simon C. Watkins, Claudette M. St Croix, Bruce R. Pitt, Li-Ming Zhang

**Affiliations:** 1 Department of Anesthesiology, University of Pittsburgh School of Medicine, Pittsburgh, Pennsylvania, United States of America; 2 Department of Environmental and Occupational Health, Graduate School of Public Health, University of Pittsburgh, Pittsburgh, Pennsylvania, United States of America; 3 Department of Surgery, University of Pittsburgh School of Medicine, Pittsburgh, Pennsylvania, United States of America; 4 Department of Cell Biology, Center for Biologic Imaging, University of Pittsburgh School of Medicine, Pittsburgh, Pennsylvania, United States of America; 5 Division of Pulmonary Allergy Critical Care Medicine, Department of Medicine, University of Pittsburgh School of Medicine, Pittsburgh, Pennsylvania, United States of America; University of Illinois College of Medicine, United States of America

## Abstract

Although a critical role for caveolae-mediated albumin transcytosis in pulmonary endothelium is well established, considerably less is known about caveolae-independent pathways. In this current study, we confirmed that cultured rat pulmonary microvascular (RPMEC) and pulmonary artery (RPAEC) endothelium endocytosed Alexa488-labeled albumin in a saturable, temperature-sensitive mode and internalization resulted in co-localization by fluorescence microscopy with cholera B toxin and caveolin-1. Although siRNA to caveolin-1 (cav-1) in RPAEC significantly inhibited albumin uptake, a remnant portion of albumin uptake was cav-1-independent, suggesting alternative pathways for albumin uptake. Thus, we isolated and cultured mouse lung endothelial cells (MLEC) from wild type and cav-1^-/-^ mice and noted that ~ 65% of albumin uptake, as determined by confocal imaging or live cell total internal reflectance fluorescence microscopy (TIRF), persisted in total absence of cav-1. Uptake of colloidal gold labeled albumin was evaluated by electron microscopy and demonstrated that albumin uptake in MLEC from cav-1^-/-^ mice was through caveolae-independent pathway(s) including clathrin-coated pits that resulted in endosomal accumulation of albumin. Finally, we noted that albumin uptake in RPMEC was in part sensitive to pharmacological agents (amiloride [sodium transport inhibitor], Gö6976 [protein kinase C inhibitor], and cytochalasin D [inhibitor of actin polymerization]) consistent with a macropinocytosis-like process. The amiloride sensitivity accounting for macropinocytosis also exists in albumin uptake by both wild type and cav-1^-/-^ MLEC. We conclude from these studies that in addition to the well described caveolar-dependent pulmonary endothelial cell endocytosis of albumin, a portion of overall uptake in pulmonary endothelial cells is cav-1 insensitive and appears to involve clathrin-mediated endocytosis and macropinocytosis-like process.

## Introduction

 Caveolae are flask-shaped membrane invaginations of the plasma membrane and caveolin-1 (cav-1, a 22-kDa protein) acts as the major structural protein and biochemical marker of caveolae [1-3]. Caveolae have been implicated in many biological processes such as endocytosis, transcytosis, and cell signaling, and they are believed to play a major role in the transport of albumin in endothelial cells. The process of endocytosis and transcytosis is the result of fission and trafficking of caveolae [4-9]. Caveolar (and cav-1) -dependent transcytosis of albumin transport in lung endothelium is of both historical [10–12] and physiological [8] significance as this aspect of cell biology has advanced [9,13-16]. In particular, from original gold-labeled albumin electron microscopy studies in intact wild type animals [17,18] to more recent investigations with similar technology in cav-1 null mice [19,20] as well as efforts in isolated cultured lung endothelial cells [21,22], a concensus has emerged regarding the importance of caveolae-dependent transcytosis of albumin in the pulmonary endothelium. The lack of uptake of gold-conjugated albumin in the pulmonary microvascular endothelium of cav-1 null, caveolae-deficient mice [14,19] underscores the role of caveolae in this process. Nonetheless, in cav-1 null mice that are devoid of caveolae, occasional invaginations with electron dense diaphragms in large vessels of the lung were observed [19]. This could account for the minor fraction of gold-labeled albumin that was taken up in fluid phase by endocytosis [18] or the preliminary observations of caveolae-independent gold-labeled albumin uptake in the lung by Predescu et al [23]. The original observation of normal albumin transport in the choroid plexus (ascribed to transcytosis) in cav-1 null mice [19] also suggests possible caveolae-independent transport of albumin. This latter observation is distinctly different from the hyperpermeability of albumin in cav-1 null mice [24,25] that is sensitive to nitric oxide synthase inhibitors and in which the role of enhanced NO biosynthesis on paracellular pathways has been elucidated in lung endothelium [21].

 Accordingly, we pursued issues of caveolae-independent transport of albumin in fixed and live cultured pulmonary endothelium. Our ultimate focus on macropinocytosis accounting for a portion of albumin uptake in pulmonary endothelium was prompted by our recent observation that another ligand, e.g. Duffy antigen receptor for chemokines (DARC), for caveolae-dependent transcytosis of chemokines, also displayed cav-1 independent endocytosis and had a pharmacological profile consistent with macropinocytosis-mediated uptake [26].

## Materials and Methods

### Cell Culture

 Rat pulmonary microvascular endothelial cells (RPMEC) were purchased from VEC Technologies (VEC Technologies, Rensselaer, NY, USA) and cultured in MCDB-131 complete medium (VEC Technologies). Rat pulmonary artery endothelial cells (RPAEC), isolated [27] and donated by Dr. Troy Stevens (University of South Alabama, Tuscaloosa, AL), were cultured in Dulbecco’s Modified Eagle Medium (DMEM, Life Technologies, Carlsbad, CA, USA) supplemented with 10% fetal bovine serum (FBS, Life Technologies, Carlsbad, CA, USA), 100 U/ml penicillin and 100 µg/ml streptomycin (Life Technologies, Carlsbad, CA, USA). RPMEC and RPAEC were incubated at 37°C in humidified atmosphere with 21% O_2_ and 5% CO_2_. Mouse lung endothelial cells (MLEC) were isolated from wild type or caveolin-1 (cav-1) null mice [28] by immunobeads coated with anti-mouse PECAM (CD31) antibody (BD Pharmingen, San Diego, CA, USA) followed by cell sorting with fluorescently-labeled acetylated-Low Density Lipoprotein (Dil-AcLDL, Biomedical Technologies Inc., Stoughton, MA, USA) uptake. MLEC were subcultured at 37°C in humidified atmosphere with 2% O_2_ and 5% CO_2_ in OPTI-MEM (Life Technologies, Carlsbad, CA, USA) supplemented with 10% FBS, 100 U/ml penicillin, 100 µg/ml streptomycin, and EC growth supplement (ENDOGRO, VEC Technologies) as previously described [29]. MLEC were studied before passage 7 and were virtually (98-100%) homogeneously positive for PECAM and uptake of Dil-AcLDL. Animal care and use were carried out in strict accordance with the recommendations in the Guide for the Care and Use of Laboratory Animals of the National Institutes of Health. The protocol was approved by the Committee on the Ethics of Animal Experiments of the University of Pittsburgh (Protocol Number: 1111998). Mouse sacrifice was performed under anesthesia and all efforts were made to minimize suffering.

### Alexa488-BSA Uptake

 Cells were plated into Lab-Tek II 4-well chamber slides (Nalge Nunc, Naperville, IL, USA) and grown until 90% confluent. After serum starvation for 4 h, cells were incubated with 50 μg/ml of Alexa488-BSA (Life Technologies, Carlsbad, CA, USA) in HBSS for indicated time at 37°C. Cells were fixed with 2% paraformaldehyde and stained with DAPI (Life Technologies, Carlsbad, CA, USA) to reveal cell nuclei and then slides were peeled off and sealed with Fluoromount-G (Southern Biotechnology Associates, Birmingham, AL, USA) under coverslips. Images were taken with Nikon Eclipse 80i fluorescence microscope (Nikon, Melville, NY, USA) and a Fluoview 500 confocal Microscope (Olympus, Center Valley, PA, USA). 

### Immunofluorescent staining of cav-1

 After incubating with Alexa488-BSA and washing with HBSS, cells were fixed with 2% paraformaldehyde for 15 min at room temperature and then permeabilized with 0.1% Triton X-100 (Sigma-Aldrich, St Louis, MO, USA) for 15 min. Blocking was performed with 20% goat serum (Sigma-Aldrich, St Louis, MO, USA) in PBS containing 0.5% BSA (Fraction V, Roche, Mannheim, Germany) for 1 h, then cells were incubated with anti-cav-1 primary polyclonal antibody (Santa Cruz Biotechnology, Santa Cruz, CA, USA) for 1 h followed by 1 h incubation with Cy3-conjugated secondary antibody (Jackson ImmunoResearch, West Grove, PA, USA). Cell nuclei were stained with DAPI (Life Technologies, Carlsbad, CA, USA) and coverslips were sealed on slides. Images were taken with Nikon Eclipse 80i fluorescence microscope and an Olympus Fluoview 500 confocal Microscope. 

### Cav-1 knockdown with siRNA

 Small interfering RNA (siRNA) was designed against the coding sequence of cav-1 cDNA (AAGAGCTTCCTGATTGAGATT) and both siRNA and sham siRNA (with scrambled sequence) were purchased from Dharmacon (Chicago, IL, USA). Transfection of siRNA (100 nM) into RPAEC was carried out by using Lipofectamine 2000 (Life Technologies, Carlsbad, CA, USA) and 72 h later Alexa488-BSA endocytosis and immunofluorescent staining of cav-1 were performed.

### Immunoblot of cav-1

 Cells were washed three times with ice-cold PBS, and then lysed on ice in SDS sample buffer (62.5 mM Tris-HCl, pH 6.8, 2% w/v SDS, 10% glycerol, 50 mM DTT, 0.01% w/v bromophenol blue). The extracts were sonicated briefly, boiled for 5 min, then centrifuged at 14,000 rpm for 5 min. Equivalent amounts of protein were separated by electrophoresis using 10% SDS-PAGE gels. The proteins were then transferred to PVDF membrane (Life Technologies, Carlsbad, CA, USA) and blocked with PBS containing 5% nonfat milk for 1 h at RT. Membranes were incubated with primary antibody against cav-1 (BD Pharmingen, San Diego, CA, USA) overnight at 4°C and HRP-conjugated secondary antibody (Santa Cruz Biotechnology, Santa Cruz, CA, USA) was used for visualization by chemiluminescence using ECL reagent (Perkin-Elmer Life Science, Boston, MA, USA). Blots were stripped with stripping buffer (Pierce, Rockford, IL, USA) and reprobed with antibody against β-actin (Sigma-Aldrich, St Louis, MO, USA). 

### Transmission electron microscopy

 Bovine serum albumin (BSA, Roche, Mannheim, Germany) was conjugated to colloidal gold particles (5 nm) as described by Baschong and Wrigley [30]. Unconjugated colloidal gold were removed by centrifugation (45,000 × g for 15 min at 4°C) of protein-gold complex, and the soft pellet was resuspended in low-salt buffer (10 mM HEPES, 1 mM KCl, 0.5 mM MgCl_2_, pH 7.5). Cells were plated into 6-well plate and grown until confluent. After serum starvation for 4 h, cells were moved to 4°C for 20 min, then incubated with Gold-labeled BSA in cold HBSS for 40 min. Cells were fixed and embedded by inverting Polybed 812-filled Better Equipment for Electron Microscopy (BEEM) capsules on top of the cells. Blocks were cured overnight at 37°C, and then cured for two days at 65°C. Monolayers were pulled off the plastic and re-embedded for cross-sectioning. Ultrathin cross-sections (60 nm) of the cells were obtained on a Riechert Ultracut E microtome, post-stained in 4% uranyl acetate for 10 min and 1% lead citrate for 7 min. Sections were viewed on a JEM 1011 transmission electron microscope (JEOL, Peabody, MA, USA) at 80 KV. Images were acquired with a side-mount AMT 2K digital camera (Advanced Microscopy Techniques, Danvers, MA, USA).

### Total Internal Reflection Fluorescence (TIRF) Microscopy

 TIRF imaging was performed as described [31]. Cells were imaged on a Nikon (Melville, NY, USA) TIRF system using a 60x (1.45 NA) optic and a Prairie (Madison, WI, USA) acousto-optic tunable filter controlled laser bench. All experiments used the 488 line on a 150-milliwatt argon source. Images were collected using MetaMorph software (Molecular Devices, Sunnyvale, CA) and a Orca II ER camera (Hamamatsu, Tokyo, Japan). Time-based series were collected and processed using MeatMorph. 

### Protocol for macropinocytosis study

 Inhibitors of macropinocytosis (Amiloride, Cytochalasin D and Gö6976) were purchased from Sigma-Aldrich (St Louis, MO, USA). RPMEC were serum starved for 4 h followed by pretreatment with amiloride (Na/H exchange inhibitor, 3 mM), Cytochalasin D (actin polymerization inhibitor, 10 µM), and Gö6976 (PKC inhibitor, 10 µM) for 30 min, then cells were incubated with Alexa488-BSA (50 µg/ml) for 15 min and fixed for imaging.

### Image quantification and statistical analysis

 Image quantification was performed with Imaris software (Bitplane Inc, South Windsor, CT). Images were thresholded and the spots function was used to define and quantify the regions of albumin uptake within the cells. Data are present as mean ± SEM. Statistical significance differences (* P < 0.05, ** P < 0.01, *** P < 0.001) were determined by t-test or one-way analysis of variance (ANOVA) followed by Tukey's multiple comparisons using Graphpad Prism ver. 5.0 (GraphPad Software, San Diego, CA, USA).

## Results

### Albumin endocytosis in rat pulmonary endothelial cells is a competitive, temperature sensitive and caveolae-dependent process

**Figure 1:**
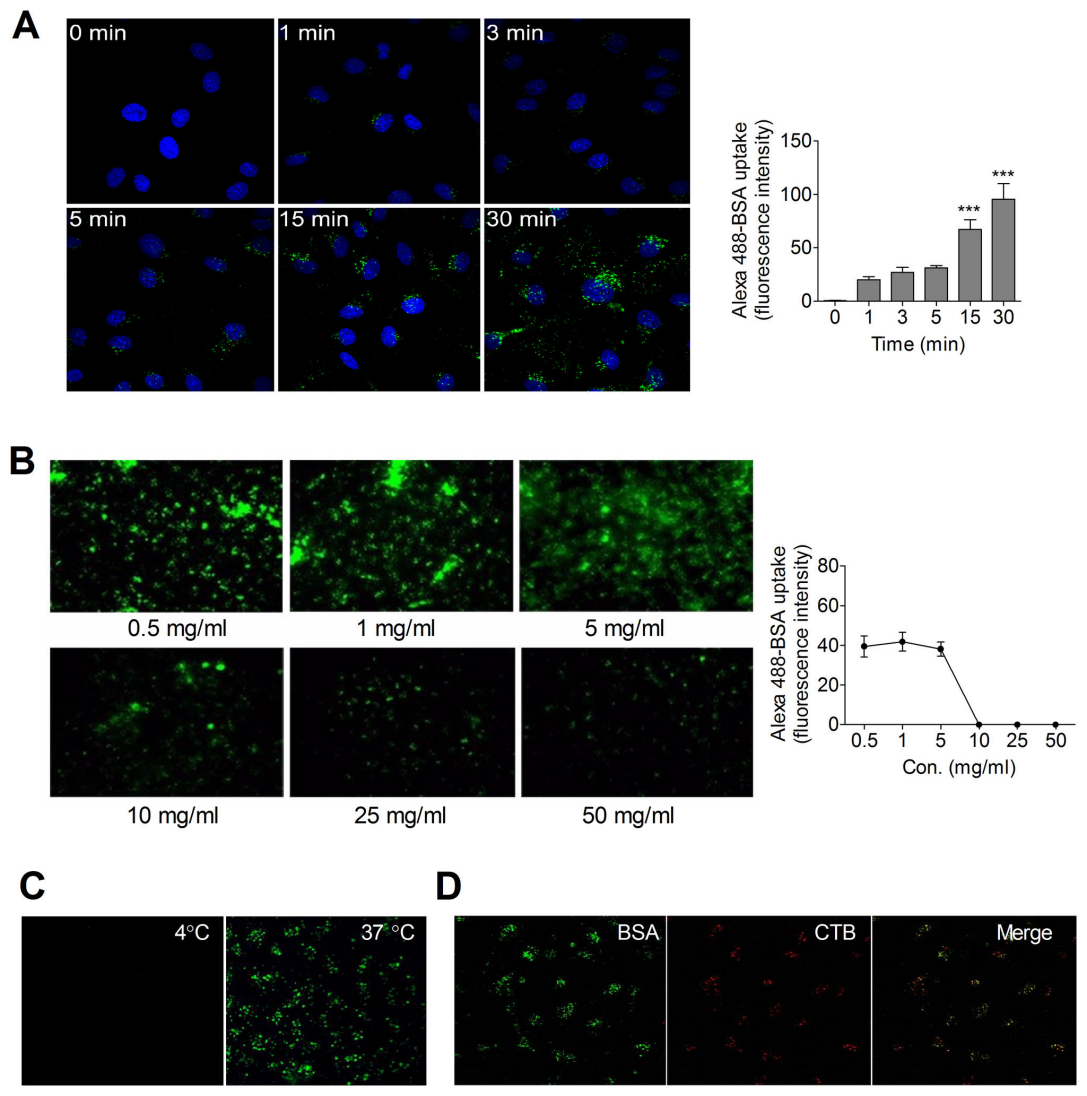
Albumin endocytosis is a competitive, temperature sensitive, and caveolae-associated process in pulmonary endothelial cells. (**A**) Time course of Alexa488-BSA endocytosis in rat pulmonary microvascular endothelial cells (RPMEC). Cells were serum starved and then incubated with Alexa488-BSA (50 µg/ml) for 0 to 30 min at 37°C. Images were taken by confocal microscopy (Olympus Fluoview 1000) at 60× (1.42 NA) magnification with 2X zoom. Representative fluorescence intensity analysis was determined by quantifying fluorescent intensity of 20-30 cells in 5 random fields using Imaris software. Values are mean ± SEM from 5 separate fields in each condition. Significant differences from control (*** P < 0.001) were determined by one-way ANOVA followed by Tukey's multiple comparisons. (**B**) Endocytosis of Alexa488-BSA by RPMEC was reduced by increasing extracellular concentrations (0.5 mg/ml - 50 mg/ml) of unlabeled BSA. Cells were serum starved for 4 h then incubated with Alexa488-BSA (50 µg/ml) in the presence of indicated concentrations of unlabeled BSA for 20 min at 37°C. Images were taken by fluorescent microscopy at 20× magnification. Representative fluorescence intensity analysis was determined by quantifying fluorescent intensity of 20-30 cells in random 5 fields with Imaris software. Values are mean ± SEM from 5 separate fields in each condition. (**C**) Alexa488-BSA endocytosis in RPMEC is temperature sensitive. Cells were incubated with Alexa488-BSA (50 µg/ml) in the presence of unlabeled BSA (0.5 mg/ml) for 20 min at 4°C and 37°C, respectively. Images were taken by confocal microscopy (60× magnification). Representative images of three independent experiments are shown. (**D**) Alexa488-BSA (green) is co-localized with Alexa555-cholera toxin subunit B (CTB, red) in RPMEC. Cells were incubated with Alexa488-BSA (50 µg/ml) and Alexa555-CTB (20 µg/ml) in HBSS containing unlabeled BSA (0.5 mg/ml) for 30 min at 37°C, cells were then washed, fixed and visualized by confocal microscopy after mounting.

 To study the capability of rat pulmonary microvascular endothelial cells (RPMEC) to uptake Alexa488-BSA, RPMEC were incubated with Alexa488-BSA (50 μg/ml) from 0 min to 30 min. Uptake was apparent at the first time point (1 min) and reached a plateau from 15-30 min (Figure 1A). A linear change of Alexa488-BSA uptake was obtained in presence of small concentrations (0.5 mg/ml - 5 mg/ml) of extracellular unlabeled BSA while almost undetectable signals were found under continuously increasing extracellular unlabeled BSA (10 mg/ml - 50 mg/ml), suggesting a competitive process occurs in albumin endocytosis (Figure 1B). Moreover, maintaining temperature at 4°C virtually eliminated Alexa488-BSA uptake (Figure 1C). After uptake, we noted (Figure 1D) significant co-localization of Alexa488-BSA and Alexa555-cholera toxin subunit B (CTB), a marker of caveolae, consistent with previous reports [32] regarding the role of caveolae in albumin uptake in pulmonary endothelial cells. After confirming (data not shown) that RPAEC manifested similar features of albumin uptake as did RPMEC, we knocked down cav-1 in RPAEC using siRNA since they are more efficiently transfected than RPMEC. 

**Figure 2:**
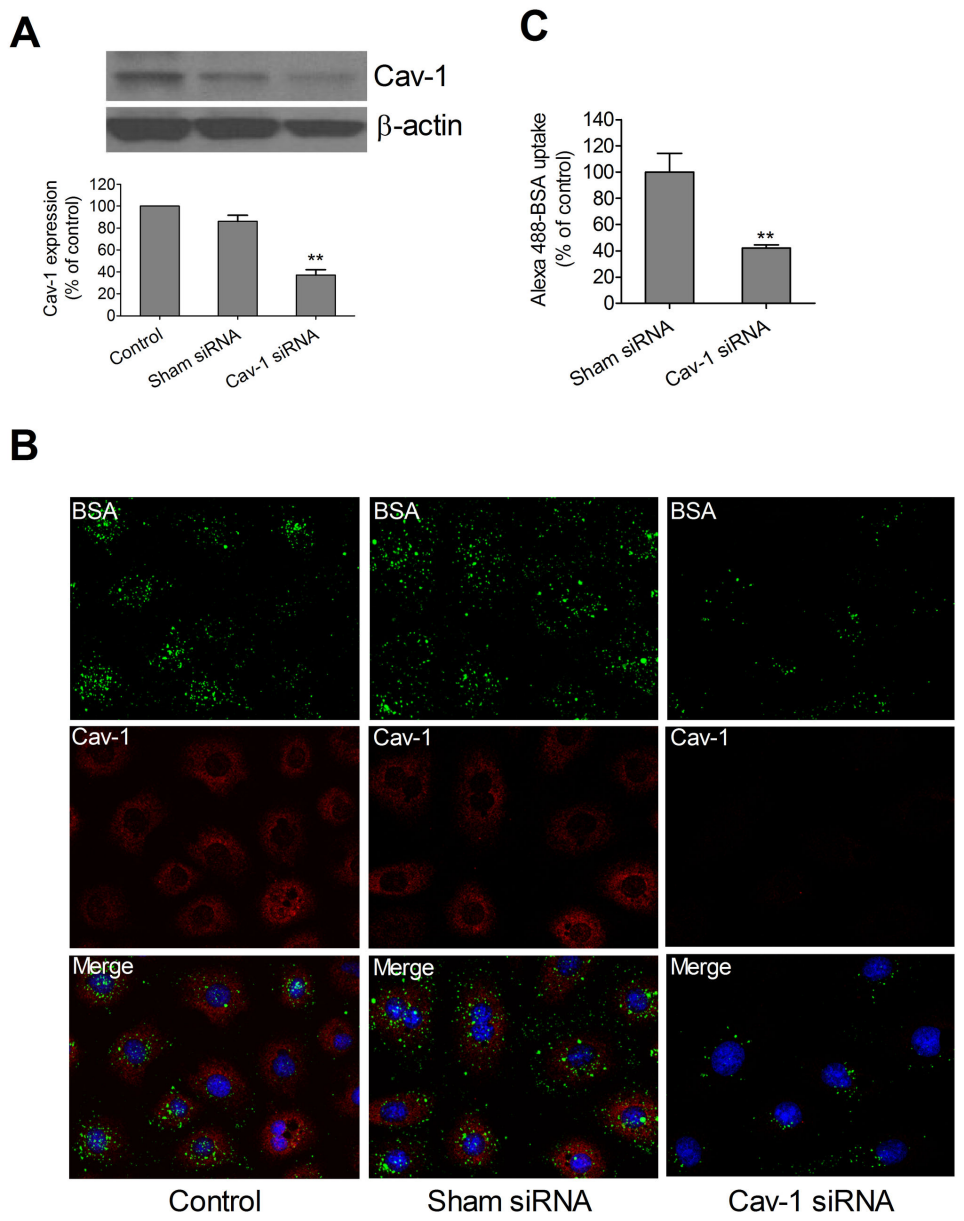
Caveolin-1 RNAi reduced Alexa488-BSA endocytosis in pulmonary artery endothelial cells. (**A**) Representative immunoblot of cav-1 expression in RPAEC treated with transfection reagent only, sham siRNA, and cav-1 siRNA is shown with β-actin as loading control. Densitometric measurement was determined by NIH Image J software. Values are mean ± SEM. Significant difference from control (** P < 0.01) was determined by one-way ANOVA followed by Tukey's multiple comparisons. (**B**) Alexa488-BSA endocytosis was reduced by siRNA to cav-1 in RPAEC. siRNA to cav-1 (100 nM) and sham siRNA (100 nM) were transfected into RPAEC with Lipofectamine 2000. After 72 h, the cells were incubated with Alexa488-BSA (50 µg/ml) for 30 min at 37°C and then fixed in 2% paraformaldehyde followed by immunofluorescence staining for cav-1. Representative confocal images (60× magnification) of three independent experiments are shown. (**C**) Representative fluorescence intensity analysis was determined by quantifying fluorescent intensity of 20-30 cells in 5 random fields with Imaris software. Values are mean ± SEM from 5 separate fields in each condition. Significant difference from control (** P < 0.01) was determined by student’s t test.

 We note that siRNA to cav-1 greatly reduced, but did not completely eliminate, immunoreactive cav-1 as detected by Western blot (Figure 2A) or immunofluorescence (Figure 2B). Uptake of non-saturating amounts of Alexa488-BSA was readily apparent in wild type or sham siRNA treated RPAEC but was greatly, but not completely, reduced (42.4 ± 2.9% persistent) in RPAEC treated with siRNA to cav-1. Alexa488-BSA co-localized with immunoreactive cav-1 in wild type and sham siRNA treated (Figure 2B). Nonetheless, because knockdown of cav-1 was incomplete (Figure 2A), it was ambiguous to credit the residual uptake of Alexa488-BSA to a caveolae-independent process and accordingly, we isolated and cultured mouse lung endothelial cells (MLEC) from wild type and cav-1 null mice. 

### Caveolae-independent albumin endocytosis presents in pulmonary endothelial cells

**Figure 3:**
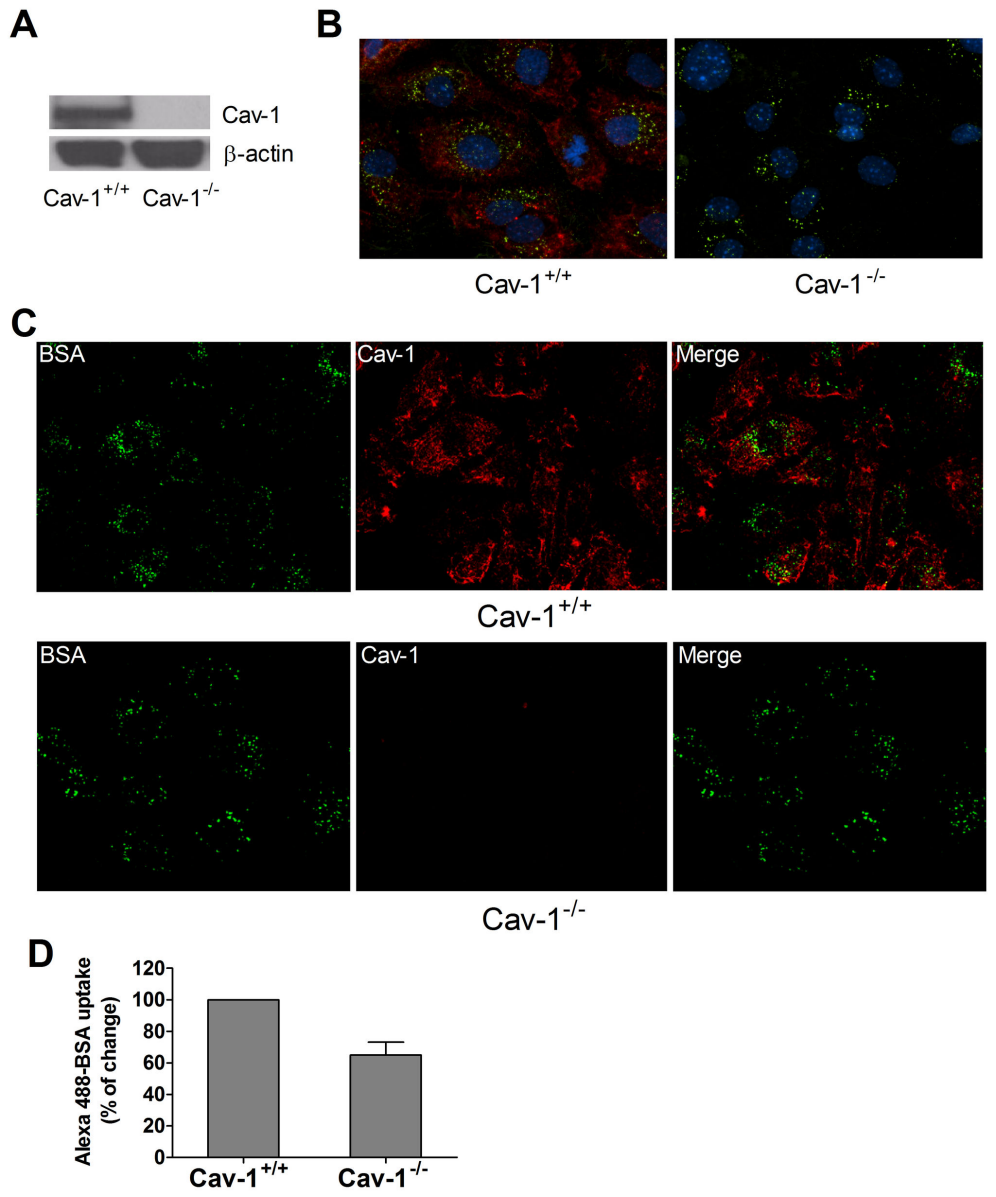
Endocytosis of Alexa488-BSA occurs in cav-1^-/-^ MLEC through a caveolae-independent pathway. (**A**) Representative immunoblot of cav-1 expression in cav-1^+/+^ and cav-1^-/-^ MLEC is shown with β-actin as loading control. (**B**) Fluorescent images and (**C**) confocal images of Alexa488-BSA (green) and cav-1 staining (red) in cav-1^+/+^ and cav-1^-/-^ MLEC are shown (60× magnification). Cells were serum starved for 4 h and incubated with Alexa488-BSA (50 µg/ml) in HBSS for 30 min at 37°C, then cells were fixed and staining of cav-1 was performed. Representative images of three independent experiments are shown. (**D**) Fluorescence intensity analysis was determined by quantifying fluorescent intensity of 20-30 cells in 5 random fields with Imaris software. Values are mean ± SEM from two independent experiments.

**Figure 4:**
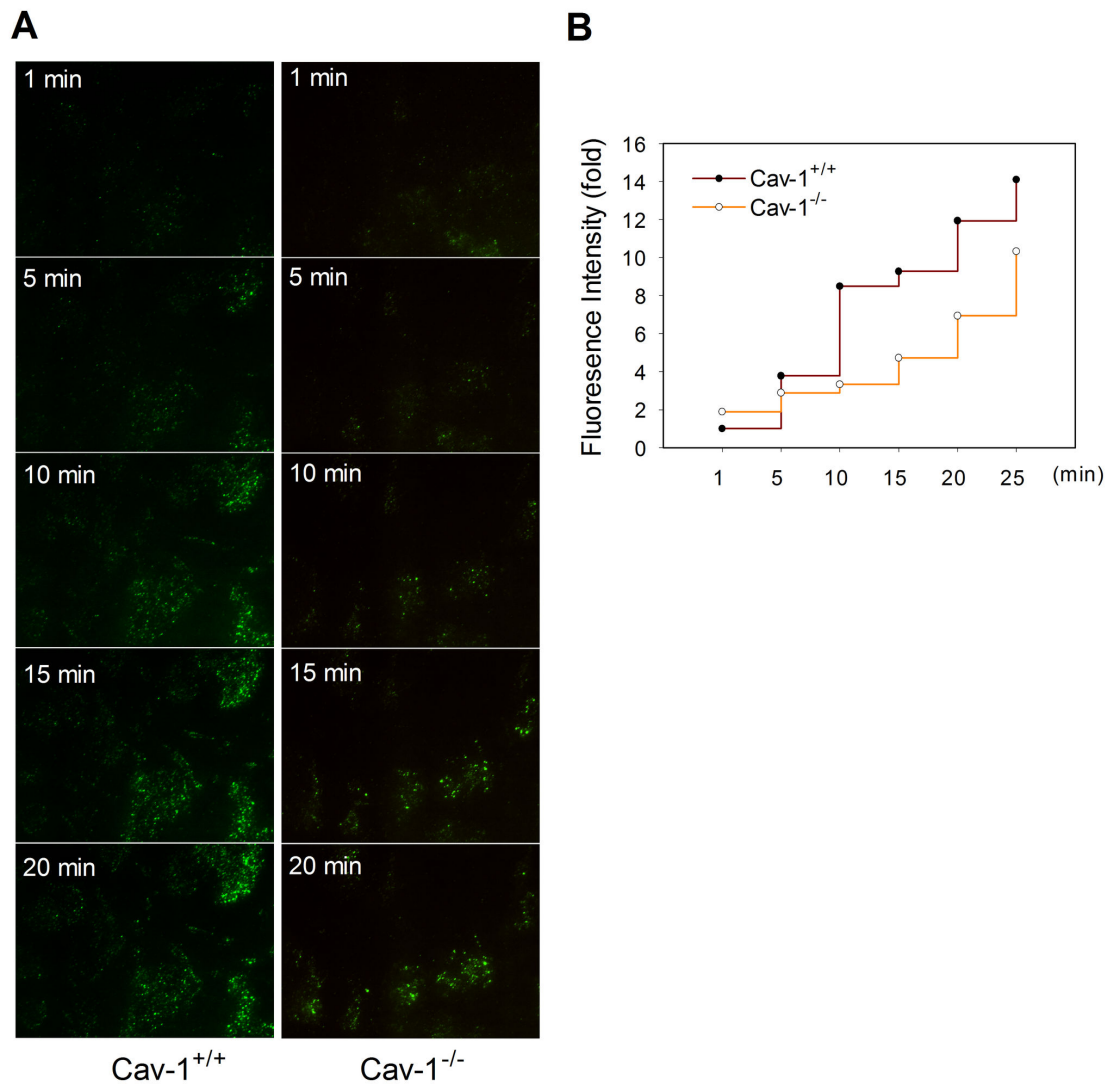
Dynamic process of Alexa488-BSA endocytosis in cav-1^+/+^ and cav-1^-/-^ MLEC. (**A**) Cav-1^+/+^ and cav-1^-/-^ MLEC were serum starved for 4 h then incubated with Alexa488-BSA (50 µg/ml) in HBSS. Fluorescence signals were recorded from 0 min to 30 min under total internal reflection fluorescence (TIRF) microscopy. Images from indicated time points (1, 5, 10, 15 and 20 min) are shown. (**B**) Quantitative analyses of Alexa488-BSA uptake in cav-1^+/+^ and cav-1^-/-^ MLEC were performed with MetaMorph software by measuring the fold change of fluorescence intensity compared to cav-1^+/+^ MLEC at 1 min after treatment.

 In contrast to wild type MLEC, MLEC from cav-1^-/-^ mice had no detectable cav-1 (Figure 3A). Similar to our experience with RPAEC (see above), there was significant uptake of Alexa488 BSA, by both widefield fluorescence (Figure 3B) and confocal (Figure 3C) microscopy in wild type MLEC. As in RPAEC treated with siRNA to cav-1, cav-1^-/-^ MLEC appeared to have a persistent remnant (65.1 ± 8.1%) of Alex488-BSA uptake compared to wild type cells (Figures 3B and 3C). To better quantify such cav-1-independent uptake, we studied the disposition of Alex488-BSA in live MLEC by TIRF (Figure 4A). Alexa488-BSA was incubated with each cell type and fluorescence signals (FITC, green) were recorded for 30 min. Although overall uptake (e.g. net fluorescence via TIRF) was greater in wild type than cav-1^-/-^ MLEC, there was still readily detectable uptake in null cells at each time point and the overall rate of uptake was comparable between the two cell types (Figure 4B). 

**Figure 5:**
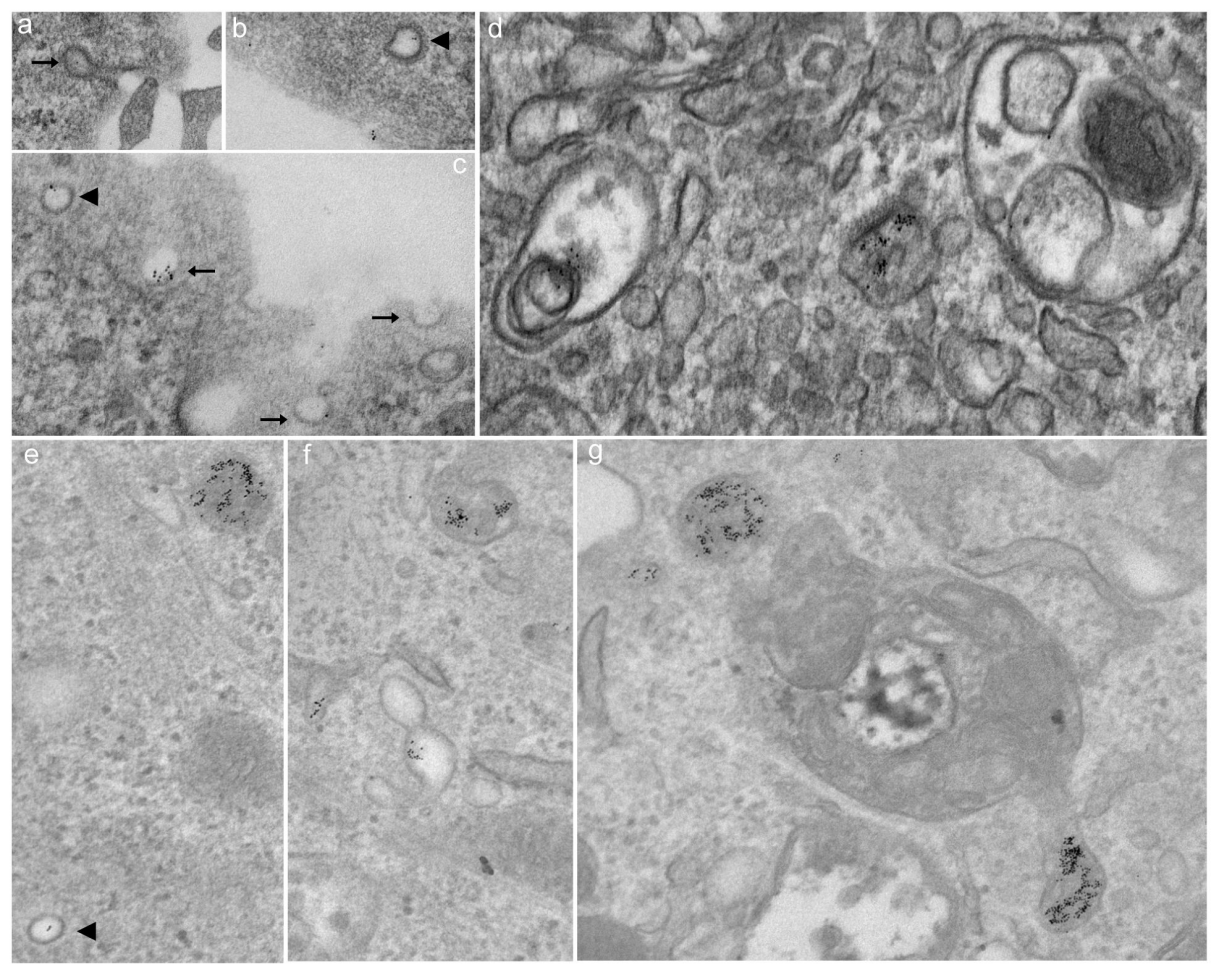
Endocytosis of gold-labeled albumin occurs in cav-1^+/+^ MLEC (a-d) through caveolae-dependent and -independent pathways but only through caveolae-independent pathway in cav-1^-/-^ MLEC (e-g). Both caveolae vesicles (black arrow) and clathrin-coated pits (black arrowhead) were positive for gold-labeled albumin (**a**-**c**) suggestive of internalization via a non-caveolin-1 dependent endocytic mechanism in cav-1^+/+^ MLEC. Although cav-1^-/-^ MLEC lack classical caveolae vesicles, there was detectable transport of gold labeled albumin to endosome and clathrin-coated pits were involved in the process (**e**-**g**). Cells were plated into a 6-well plate and grown until confluent. After serum starvation for 4 hr, cells were incubated with gold-labeled BSA in cold HBSS and moved to 4°C for 1 hr followed by warming at 37°C for up to 40 min. Cells were fixed and images were taken by transmission electron microscopy (80000× magnification).

In addition, we compared the uptake of gold-labeled albumin in cav-1^+/+^ MLEC with that of cav-1^-/-^ MLEC by using transmission electron microscopy. Although cav-1^-/-^ MLEC lack classical “Ω” shape caveolae vesicles, there was detectable transport of gold-labeled albumin through caveolae-independent process including clathrin-coated pits by 40 minutes, and electron dense material was associated with endosomes; Interestingly, in addition to caveolae-mediated albumin uptake, we also observed clathrin-mediated albumin transport in cav-1^+/+^ MLEC (Figure 5).

### Macropinocytosis is involved in albumin endocytosis in pulmonary endothelial cells

**Figure 6:**
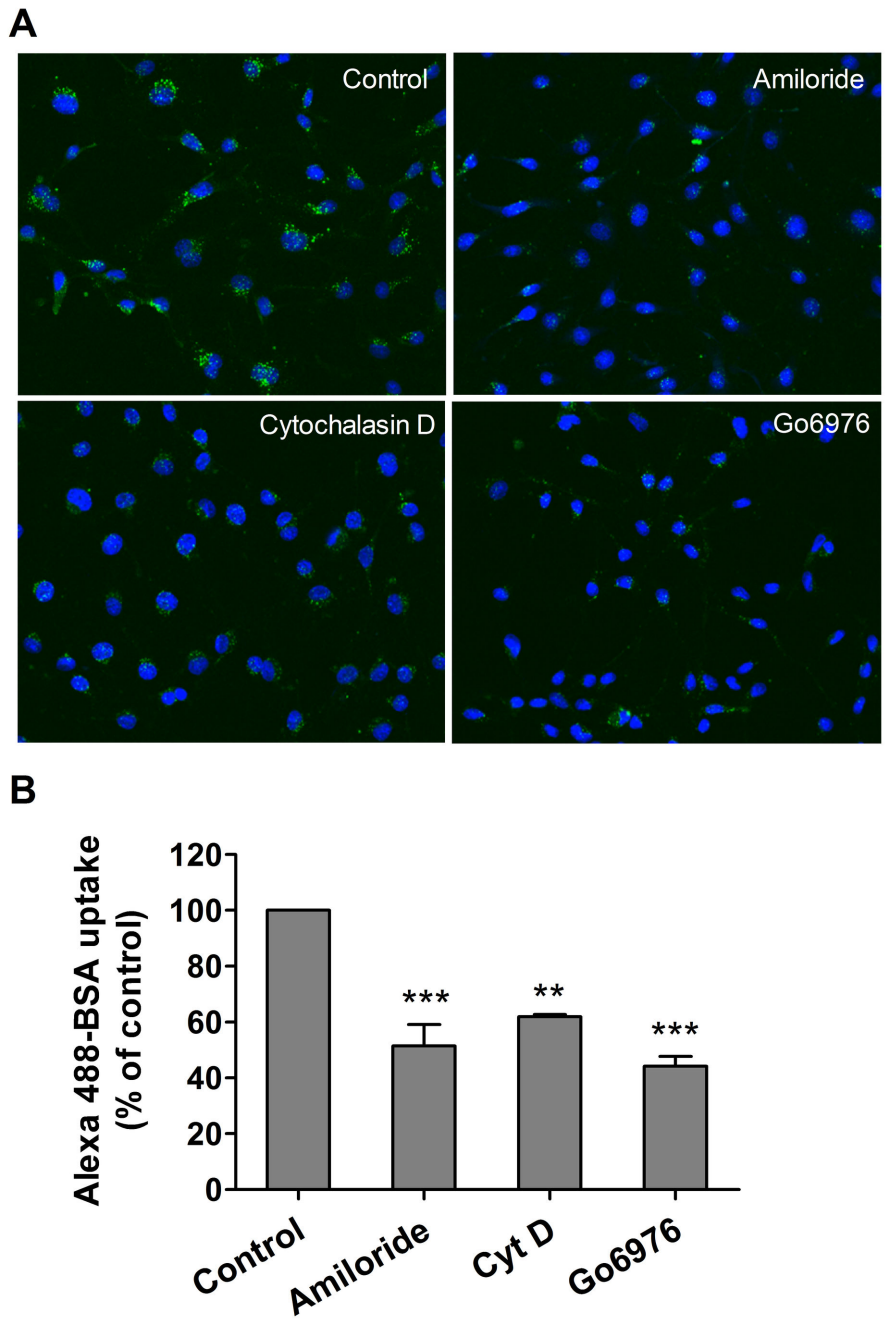
Inhibition of macropinocytosis prevents Alexa488-BSA uptake by rat pulmonary endothelial cells. (**A**) RPMEC were serum starved for 4 h followed by pretreatment with various inhibitors of macropinocytosis for 30 min including amiloride (Na/H exchange inhibitor, 3 mM), Cytochalasin D (actin polymerization inhibitor, 10 µM), Gö6976 (PKC inhibitor, 10 µM), then cells were incubated with Alexa488-BSA (50 µg/ml) for 15 min and fixed with 2% paraformaldehyde for imaging. Images were taken by confocal microscopy (40× magnification). Representative images from three independent experiments are shown. (**B**) The fluorescence intensity was determined by Imaris software with data are present as mean ± SEM from three independent experiments. Significant differences from control (** P < 0.01, *** P < 0.001) were determined by one-way ANOVA followed by Tukey's multiple comparisons.

 The dispensability of cav-1 for at least a portion of albumin uptake was reminiscent of our recent experience with another ligand, Duffy antigen receptor for chemokine, and its role in chemokine internalization and transport across endothelium, previously assumed to involve a process of caveolar-dependent transcytosis [26]. Accordingly, we tested the drug sensitivity of a potential macropinocytic pathway and noted that uptake of Alexa488-BSA in RPMEC was significantly decreased by amiloride (inhibitor of Na/H exchange) and Gö6976, a protein kinase C inhibitor. In addition, cytochalasin D, an actin polymerization inhibitor also reduced uptake (Figure 6). We confirmed that albumin uptake was amiloride sensitive in both cav-1^+/+^ and cav-1^-/-^ MLEC since 70% of albumin uptake was reduced in cav-1^-/-^ cells while only 50% reduced in cav-1^+/+^ cells (data not shown). Collectively these data are consistent with macropinocytosis-like process accounting for a portion of albumin uptake in both rat and mouse endothelium. 

## Discussion

 Transcytosis, or the transport of molecules across cells, is a fundamental aspect of cell biology and maintains tissue homeostasis and normal organ function [9]. From a historical and physiological basis, transcellular transport of albumin across pulmonary endothelium has been one of the most studied of such systems. In particular, initial studies with gold-labeled albumin and transmission electron microscopy in the pulmonary circulation suggested a dynamic process that coupled endocytosis to exocytosis and potential directional movement of macromolecules [17] leading Simionescu and colleagues to coin the term transcytosis. Further structural and functional studies led to identification of the role of caveolae (and cav-1) in this process and ultimately led several groups [19,33,34] to genetically ablate cav-1. Their collective experience in these cav-1 knock out mice clearly demonstrated a lack of caveolae biogenesis and an association with greatly depressed accumulation of albumin. The compensatory hyperpermeability to albumin in cav-1 null mice has been ascribed to increased NO biosynthesis and the molecular details accounting for increased paracellular transport in the absence of transcytosis have been delineated in vivo [24,25,35] and in cell culture [21]. As such, pulmonary endothelium has been a useful prototype to advance fundamental aspects of cell biology and contribute to the ongoing debate [36] of relative contributions of transcytosis [9,37] and paracellular [38] pathways or both [39] to determinants of vascular permeability. Further molecular details of the roles of dynamin, Rac and actin in co-regulation of transcytosis and paracellular transport were recently described in cultured human microvascular endothelium of dermal origin [40] which is an extension of critical roles of dynamin and actin cross-linking protein Filamin A in albumin uptake demonstrated previously although this likely does not distinguish caveolae- from clathrin-mediated endocytosis [22,41]. 

 Considerably less is known regarding cav-1-independent pathways [9]. From the original report of Drab et al [19], there were descriptions of invaginations with electron dense diaphragms within endothelium of large vessels of cav-1 null mice and a persistent uptake of albumin in the choroid plexus. In this latter instance, albumin uptake has been proposed to be a caveolae-dependent process [42] as it is in cultured astrocytes [43]. A preliminary study indicated that vesicular structures within endothelium, but without cav-1 (or clathrin) coats, may be capable of functioning in transport of gold albumin [23]. More recently [44], knockdown of a critical adapter/scaffold protein, Intersectin-1s, led to impaired biogenesis of caveolae and upregulation of a potential alternative endocytic pathway in pulmonary endothelial handling of gold labeled albumin. Whether cav-1-independent vesiculo-vacuolar organelles in systemic venules are important in transcytosis is also unclear. They persist, however, in the cav-1 null mouse [45]. Several studies have noted that siRNA to cav-1 treatment of cultured endothelium or astrocytes results in partial decreases in albumin uptake (approximately 50-60%). Silencing of cav-1 was not complete [43,46] and thus the possibility of a coexisting alternative pathway remained ambiguous. This is somewhat reminiscent of observations in the current study in which albumin uptake (and cav-1) was only partially inhibited in cav-1 siRNA treated cells (Figure 2A and 2B). Indeed such ambiguity prompted us to move from rat to mouse lung endothelial cells that were subcultured from cav-1 null mice. With ambiguities of partial depletion aside, we still noted residual (~ 65%) cav-1 independent uptake of albumin. This was apparent by epifluorescence and confocal microscopy (Figure 3B and 3C) of fixed cells as well as via TIRF analysis in live MLEC (Figure 4). In the latter case, the overall magnitude of uptake was less in cav-1 null cells but the rate of uptake appeared similar to that noted in wild type cells. Cav-1 null MLEC took up gold-labeled albumin likely through cav-1-independent pathway(s) including clathrin-mediated endocytosis, and the disposition appeared to be in endosomes (Figure 5). 

 A number of other more elemental insights into caveolae (and clathrin)-independent transport have emerged. In particular, cholera toxin binding (CTB) subunit, a traditional biomarker of caveolae-dependent processes, utilized a cav-1 independent, uncoated tubular or ring shaped structure containing GPI-anchored proteins and fluid phase markers that mediated CTB uptake in wild type and cav-1 null mouse embryonic fibroblasts [47]. A simian virus 40 activated endocytic pathway in human hepatoma 7 (deficient in cav-1) and embryonic fibroblasts from cav-1 null mice was described that did not involve either clathrin-coated pit or caveolae [48]. Most relevant to the current study was our recent observation that Duffy Antigen Receptor for Chemokines (DARC) mediates chemokine endocytosis through a macropinocytosis-like, caveolae-independent process in endothelial cells [26]. Even though DARC co-localized to cav-1 and DARC-mediated chemokine internalization was cholesterol sensitive, internalization of ^125^I-CXCL1 was independent of cav-1 (and clathrin and flotillin-1). A macropinocytosis-like process appeared to account for such internalization and chemokine transport. These results prompted us to determine if macropinocytosis might contribute to uptake of another ligand, albumin, in cultured endothelium. We noted that uptake of Alexa488-BSA in RPMEC was significantly decreased by amiloride (inhibitor of Na/H exchange), Gö6976 (protein kinase C inhibitor), and cytochalasin D (actin polymerization inhibitor) (Figure 6). Such a pharmacological profile is consistent with a macropinocytosis-like pathway contributing to albumin uptake in cultured rat pulmonary endothelium and may account for the persistent uptake of albumin noted in the presence of siRNA to cav-1 in RPAEC (Figure 2B) or in MLEC from cav-1 null mice (Figure 3, 4, 5). It is possible that upregulation of this pathway may have occurred secondary to cav-1 silencing as pretreatment of amiloride depleted most (~70%) albumin uptake in cav-1^-/-^ MLEC (data not shown). Our results are consistent with a study by Cheng et al with respect to enhanced fluid phase endocytosis after cav-1 silencing [49]. In addition, Muro et al [50] identified a novel endocytic mechanism to regulate the internalization of anti-ICAM-1 and anti-PECAM-1 conjugates by endothelial cells which is distinct from caveolae-mediated uptake or clathrin-mediated endocytosis. This CAM-mediated endocytotic process was sensitive to amiloride and PKC inhbitors [50] and thus shared features of macropinocytosis as we have described in our current and previous [26] studies. We did not examine requisite role of dynamin-2 and other signaling and cytoskeletal protein dependencies that led Muro et al [50] to distinguish CAM-mediated endocytosis from macropinoyctosis.

 In the current study, we show that cultured rodent pulmonary endothelium (rat and mouse, pulmonary arterial and microvascular) transport albumin by cav-1-dependent and -independent processes. In addition to the well documented caveolae-associated transcytosis, at least secondary pathways including macropinocytosis and clathrin-mediated endocytosis are apparent in these cells. A macropinocytosis-like process may be compensatory and reminiscent of cav-1-independent fluid phase transport of CTB [47] or albumin [49]. It bears some similarity to the macropinocytosis pathway noted by us for DARC mediated chemokine transcytosis [26]. In transmission electron microscopy/gold-labeled albumin studies, we noted that albumin is initially delivered to endosomes in cav-1^-/-^ MLEC via cav-1-independent pathways involving clathrin (Figure 5) which may partly explain why albumin uptake was not completely eliminated (~30% remnant) by amiloride (inhibitor of macropinocytosis) in cav-1^-/-^ MLEC. It is not apparent whether drug sensitive profile of macropinocytosis of albumin was coupled to a shuttling and exocytotic egress. The contribution of potential artifacts secondary to studying cultured endothelial cells in a monolayer on plastic dishes makes it ultimately important to determine the functional consequences of cav-1-independent albumin uptake in vivo, and whether it contributes to potential regional differences (large vs small vessel) and organ (brain vs lung, etc) heterogeneity of albumin transport [9,51] as well as affecting determinants of transvascular protein transport [36,38]. 
